# Dietary Folate and Cofactors Accelerate Age-dependent *p16* Epimutation to Promote Intestinal Tumorigenesis

**DOI:** 10.1158/2767-9764.CRC-23-0356

**Published:** 2024-01-19

**Authors:** Li Yang, Robert C. Peery, Leah M. Farmer, Xia Gao, Yiqun Zhang, Chad J. Creighton, Lanjing Zhang, Lanlan Shen

**Affiliations:** 1 USDA Children's Nutrition Research Center, Department of Pediatrics, Baylor College of Medicine, Houston, Texas.; 2 Department of Molecular and Cellular Biology, Baylor College of Medicine, Houston, Texas.; 3 Dan L. Duncan Comprehensive Cancer Center Division of Biostatistics, Baylor College of Medicine, Houston, Texas.; 4 Department of Medicine and Human Genome Sequencing Center, Baylor College of Medicine, Houston, Texas.; 5 Department of Pathology, Princeton Medical Center, Plainsboro, New Jersey.; 6 Department of Chemical Biology, Earnest Mario School of Pharmacy, Rutgers University, Piscataway, New Jersey.

## Abstract

The extent to which non-genetic environmental factors, such as diet, contribute to carcinogenesis has been long debated. One potential mechanism for the effects of environmental factors is through epigenetic modifications that affect gene expression without changing the underlying DNA sequence. However, the functional cooperation between dietary factors and cancer-causing epigenetic regulation is largely unknown. Here, we use a mouse model of age-dependent *p16* epimutation, in which the *p16* gene activity is directly controlled by promoter DNA methylation. We show *p16* epimutation is modulated by folate and cofactors in dietary supplementation, which leads to increased colon cancer risk. Importantly, our findings provide functional evidence concerning the safety of folate fortification in the general population.

**Significance:**

Our study demonstrates that dietary folate and cofactors modulate tumor-suppressor gene methylation to increase intestinal tumorigenesis. Our findings highlight the need for monitoring the long-term safety of folate fortification in high-risk individuals.

## Introduction

Mammalian one-carbon (1C) metabolism, which provides the methyl groups for synthesis and methylation of DNA, is highly dependent on dietary factors such as folate and other B vitamins (1). Notably, over the past 25 years, the general U.S. population has been exposed to a significant increase in folate intake, due to both an increase of the allowable supplement dosage by the FDA and mandatory food fortification of folic acid which is a synthetic form of folate to prevent neural tube birth defect in pregnant women (2). In addition, a high folate diet has been used in clinical trials to prevent age-related chronic diseases, including colon cancer. However, in several large, randomized studies, individuals who supplemented folate were as likely to develop cancer and precancerous tumors as placebo controls (3, 4). Furthermore, clinical studies suggest long-term supplementation with folate and vitamin B12 may alter gene-specific DNA methylation in aging populations (5–7). However, to date, no study has directly evaluated the epigenetic effects of dietary intervention on colon cancer outcomes.

To perform functional studies, we developed a mouse model of colon cancer that replicates two common genetic and epigenetic events observed in human colorectal cancers: *Apc* mutation and *p16* epimutation (8, 9). Our previous work showed that age-dependent *p16* epimutation cooperates with mutant *Apc* to modulate the tumor immune microenvironment and accelerate intestinal tumorigenesis, thus directly linking colorectal cancer development to a defined epigenetic driver event (8). The current study aims to determine whether specific nutrients related to 1C metabolism can modulate *p16* epimutation and increase susceptibility to colon cancer.

## Materials and Methods

### Mice and Diets

All experiments were performed in the *Apc^Min^^/+^*; *p16^cis/^^cis^* mice carrying a combined *Apc* mutation (RRID: MGI:1856318) and *p16* epimutation (RRID: MGI: 5660270). The *Apc^Min^^/+^*; *p16^cis/^^cis^* mice were generated previously (8). Both control NIH-31 (TD.95262) and supplemented NIH-31 (TD.10769) diets were made by Envigo-Harlan and kept in the refrigerator upon arrival. All animal research was approved by the Baylor College of Medicine Animal Care and Use Committee.

### Metabolomic Analysis

Untargeted metabolomics analysis by LC/MS was performed as described previously with minor modifications (10). Briefly, all mice used were at 15 weeks of age (*n* = 7–9 per group). A total of 6 mg of liver and tumor samples and 10 µL of serum, were collected and used for polar metabolite extraction. A Vanquish Horizon ultra-high-performance liquid-chromatography system coupled with Orbitrap Exploris 480 mass spectrometer (Thermo Fisher Scientific) was used for metabolite separation and detection.

### Histologic Analysis

Colon tissues from 15 weeks old mice (*n* = 4 per group) were fixed in 4% paraformaldehyde. Tumor sections were stained with hematoxylin and eosin and IHC antibodies: Ki-67 (Thermo Fisher Scientific, catalog no. MA5-14520, RRID: AB_10979488, 1:100), CD45 (Thermo Fisher Scientific, catalog no. 14-0451-82, RRID: AB_467251, 1:200), CD68 (Cell Signaling Technology, catalog no. 97778, RRID: AB_2928056, 1:200), F4/80 (Cell Signaling Technology, catalog no. 70076, RRID: AB_2799771, 1:200). All antibody staining was performed overnight at 4°C, followed by incubation with either anti-rabbit or anti-goat secondary antibody. Slides were developed using a 3,3′-Diaminobenzidine (DAB) kit (Thermo Fisher Scientific, catalog no. 34065), and nuclei were counterstained with hematoxylin. Images were captured using Olympus imaging software, cellSens standard version 2 (RRID: SCR_014551). The average number of Ki-67^+^ cells per mm^2^ of tumor area was counted in individual tumors from at least five randomly selected microscopic field at high magnification (40×). The methods for assessing of tumor-infiltrating lymphocytes (TIL) were determined on the basis of the recommendation provided by the International TILs Working Group (11). The average number of CD45^+^, CD68^+^, and F4/80^+^ TILs was counted and analyzed separately in either intratumoral or tumor-surrounding stromal compartments.

### Single-cell RNA Sequencing Analysis

Colon tumors and adjacent normal tissues were collected and processed on the same day for single-cell RNA sequencing (scRNA-seq) as described previously (8). Prior to conducting the sequencing, samples were screened for high quality (viability >90% and live cells >10,000). After passing the quality control, we were able to analyze 10 tumors from 3 supplemented mice (19 to 24 weeks old). Cell Ranger pipeline (10x Genomics, default settings, v3.1.0, RRID:SCR_017344) was used to process the raw sequencing files. Unsupervised discovery of cellular clusters was performed in R using Seurat v4.1.0 R package (RRID:SCR_016341; ref. 12). Individual clusters were identified on the basis of proximity of the clusters in a t-distributed stochastic neighbor embedding (tSNE, RRID:SCR_024305) or uniform manifold approximation and projection (UMAP) (RRID:SCR_018217) plot and expression patterns for key marker genes. Cell clusters were annotated on the basis of expression of marker genes, as drawn from the literature and published cellular marker gene lists related to immune cells (13) and small intestinal epithelium (14). In addition, to detect the subpopulations of normal and tumor epithelial cells, we included the previously published scRNA-seq data on normal colonic mucosa (8).

### Colonic Tumor Organoid Culture

Detailed procedures to grow and maintain tumor organoids from mouse colons were described previously (8). Tumorigenic organoids were cultured in NE medium containing Noggin and EGF, and without Wnt-3A and R-spondin (provided by Texas Medical Center Digestive Diseases Center Core). We used the organoids at passage 2 for further analysis.

### DNA Methylation Analysis

For quantitative DNA methylation measurements, bisulfite pyrosequencing was performed using a PyroMark Q48 instrument (Qiagen) as described previously (8, 9). Primer sequences and sequencing assays are available upon request.

### Statistical Analysis

GraphPad Prism (RRID:SCR_002798) was used to perform statistical analyses. Significance of differences were determined by Kaplan–Meier survival analysis, log-rank test, and two-tailed Student *t* test. *P* values ≤0.05 were considered statistically significant.

### Data Availability

All scRNA-seq data have been uploaded to Gene Expression Omnibus and are available at the accession number GSE214032. The other data are available upon request from the corresponding author.

## Results

**FIGURE 1:**
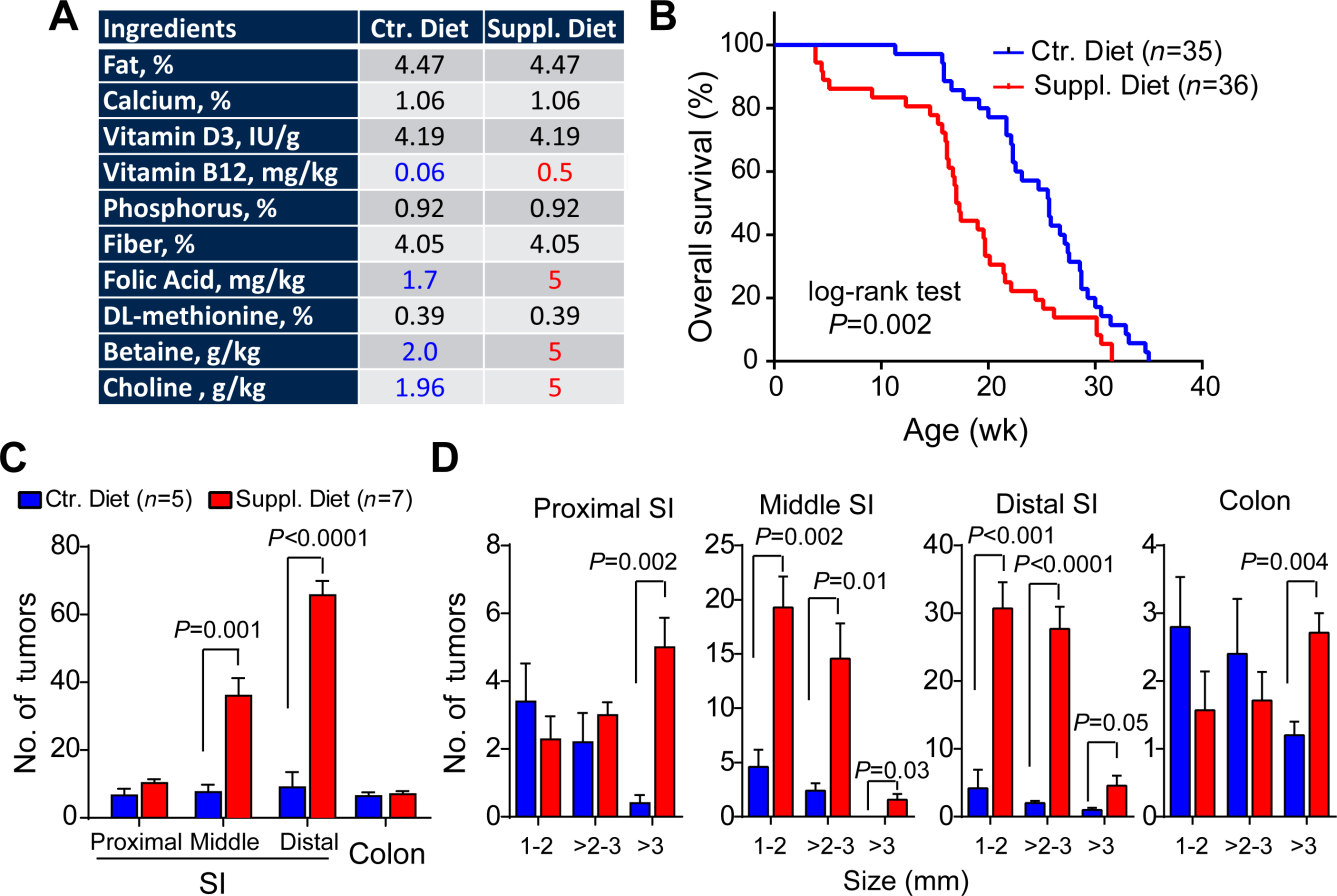
Dietary supplementation of folate and cofactors drives an aggressive intestinal tumor phenotype in mice carrying combined *Apc* mutation and *p16* epimutation. **A,** Diet formulations are shown for both control (ctr.) and supplemented (suppl.) diets. The supplemented ingredients are denoted in blue and red font. **B,** Compared with controls, mice administered a supplemented diet exhibit significantly shorter overall survival. Survival was compared using the Kaplan–Meier method and *P* values were determined by a log-rank test. **C,** Mice on the supplemented diet display a significantly increased number of tumors in the middle and distal regions of small intestines (SI) at 15 weeks of age. **D,** Mice on a supplemented diet develop substantially more large tumors based on maximum diameter throughout the entire intestinal tracts at 15 weeks of age. All data are presented as mean ± SEM. *P* values were determined by two-tailed Student *t* tests.

The diet compositions are summarized in Fig. 1A. To mimic the food fortification program, we provided the diet to female mice before conception, during pregnancy, and in offspring throughout life. We used an amino acid–defined NIH-31 diet supplemented with extra choline, betaine, folic acid, and vitamin B12, based on previous studies showing that maternal supplementation increases locus-specific DNA methylation in offspring (15, 16). More importantly, the modest amount of supplemented folic acid (∼3-fold increase) is equivalent to FDA-mandated folate fortification (17). Of note, in our previous study (8), we used a LabDiet (5V5R) that varied substantially in macronutrients and micronutrients from the current NIH-31 diet (Supplementary Table S1). Mice carrying both *Apc* mutation and *p16* epimutation were assigned randomly to either the control NIH-31 diet (*N* = 35) or supplemented NIH-31 diet (*N* = 36). As shown in Fig. 1B, we found mice on the supplemented diet had a significantly shortened overall survival compared with those on the control diet (*P* = 0.002, log-rank test). To determine whether dietary supplements affect neoplastic transformation at an early stage, we assessed whole bowel tumor formation from additional cohorts of mice at 15 weeks of age (*N* = 5–7 per group) using a dissecting microscope. Consistent with the survival analysis, we detected more tumors within the middle and distal regions of the small intestine in supplemented mice relative to controls (Fig. 1C). Interestingly, in both small intestines and colons, the supplemented mice displayed a significantly higher number of large tumors (diameter >3 mm) compared with controls (*P* ≤ 0.05 by two-tailed Student *t* tests, Fig. 1D). Moreover, the average size of colon tumors from supplemented mice was above 3 mm, significantly higher than those from the control mice (∼2 mm, *P* = 0.006; Supplementary Fig. S1). Collectively, these data demonstrate dietary folate and cofactors exacerbate the tumor phenotype in our mouse model of colon cancer.

**FIGURE 2:**
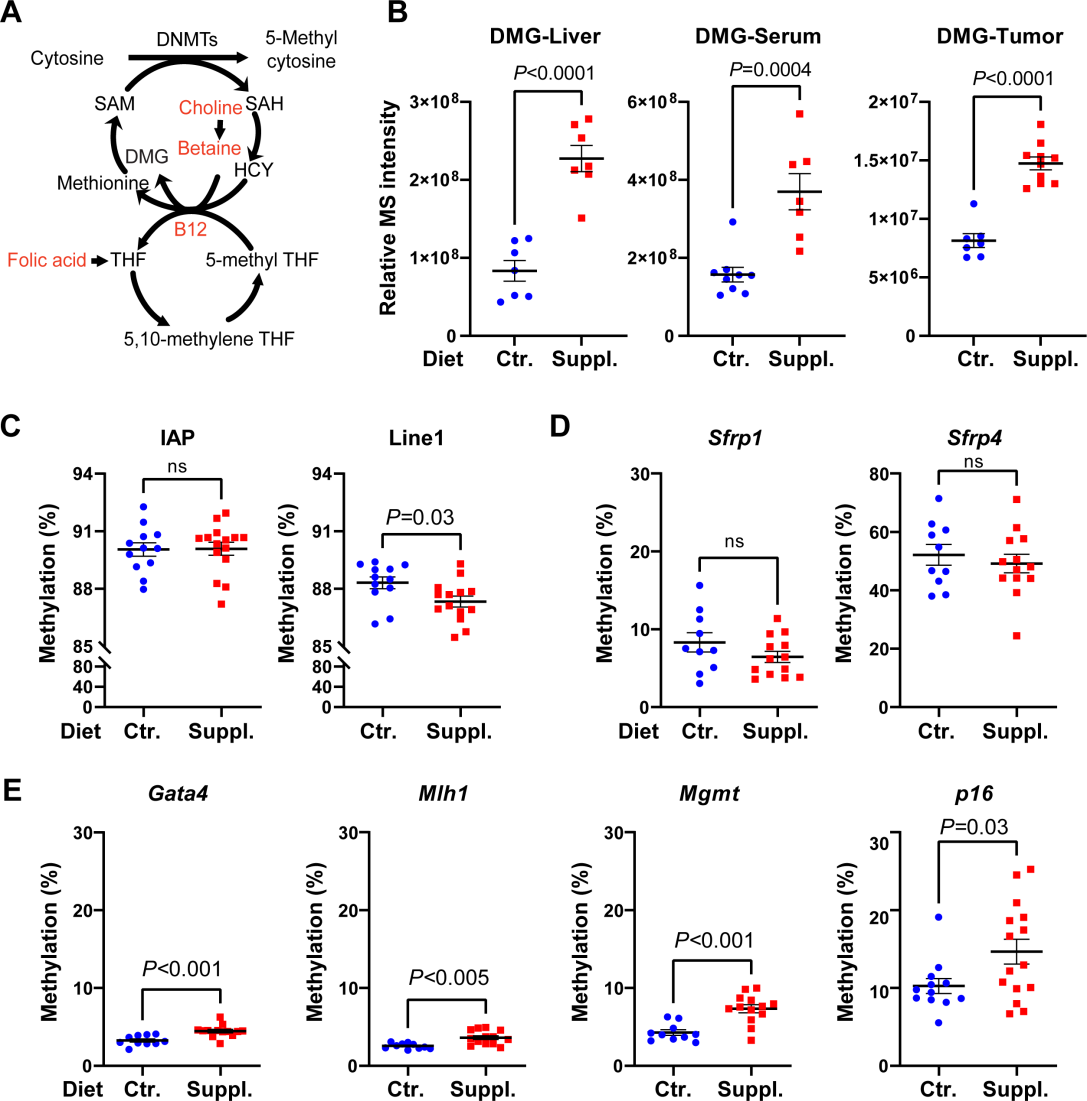
Dietary supplementation increases methyl-donating metabolites that promote *p16* promoter methylation in colon tumors. **A,** A simplified scheme of the 1C metabolism with DMG as an intermediate metabolite in the 1C transfer cycle, which is available for further methyl donation. Dietary supplements are highlighted in red. **B,** Dietary folate and cofactors significantly increase the concentrations of DMG across all tissues analyzed. **C,** DNA methylation status at repetitive elements. **D,** Promoter DNA methylation analysis of two Wnt antagonist genes. **E,** Promoter DNA methylation analysis of other colorectal cancer–related tumor suppressor genes. All data are presented as mean ± SEM. *P* values were determined by two-tailed Student *t* tests. DMG: N,N-dimethylglycine; ns: not significant; SAH: S-adenosyl-L-homocysteine;.

To gain further insights into the cause-and-effect relationship, we performed comparative metabolomic analysis across a panel of samples including liver, serum, and tumor. On the basis of fold changes (>1.5 or <0.67) and *P* values (≤0.05), we detected the most differentially expressed metabolites in the liver (57/78 were upregulated with dietary supplementation), and to a lesser extent in the serum (23/25 were upregulated with dietary supplementation) and tumors (8/16 were upregulated with dietary supplementation; all data are included in Supplementary Tables S2–S4). As predicted, because liver is the major metabolic organ in the body, we detected significantly increased metabolites in the 1C metabolism with food supplementation, including choline, methionine, S-adenosyl-L-homocysteine (SAH), and N,N-dimethylglycine (DMG; Supplementary Fig. S2A). Of particular interest, DMG is a metabolic intermediate in the 1C transfer cycle which has the potential to maintain a high state of transmethylation (Fig. 2A). Indeed, across all samples analyzed, the supplemented diet led to an approximately 3-fold increase in the abundance of DMG relative to controls (Fig. 2B). The ratio of S-Adenosyl-methionine (SAM) to SAH is also a well-established marker to indicate the likelihood of hypermethylation. Consistently, we saw increased SAM/SAH ratios in the tumors from supplemented mice (Supplementary Fig. S2B). Together, our data confirm that, in response to diet, an enhanced 1C metabolic pathway contributes to tumor growth.

Next, we asked whether the epigenetic effects of diet could explain the observed tumor phenotype for mice on the supplemented diet. *p16* epimutation is well known for promoting cell cycle progression through the p16-cyclin D-CDK4/6-RB pathway. In addition, we previously showed that colon tumors with accelerated *p16* epimutation are characterized by extensive tumor-infiltrating immune cells throughout tumor progression (8). Therefore, we performed histologic assessments of colon tumors from the cohorts of 15 weeks old mice. To quantify the rate of tumor cell proliferation, we used IHC staining of Ki-67. To determine the number of TILs, we used IHC staining of CD45 (pan-leukocyte marker), CD68 (pan-macrophage marker), and F4/80 (monocyte-macrophage marker). Consistent with the aggressive tumor phenotype, we found that Ki-67–positive tumor cells were significantly higher under dietary supplementation compared with controls (*P* < 0.05 by two-tailed Student *t* tests; Supplementary Fig. S3A). Interestingly, comparing the two groups, the infiltrating CD45^+^ leukocytes were similar in both intratumoral and surrounding stromal areas (Supplementary Fig. S3B). In contrast, colon tumors from the supplemented mice contained significantly more macrophages in the tumor stroma relative to those on the control diet (*P* values for CD68 and F4/80 are 0.01 and 0.03, respectively, by two-tailed Student *t* tests; Supplementary Fig. S3B). Furthermore, scRNA-seq analysis allows a high-resolution characterization of tumor-associated macrophages within the supplemented colon tumors (Supplementary Fig. S4A and S4B). Consistently, we found the remarkable infiltration of immune cells (Supplementary Fig. S4C) and the majority of non-tumor epithelial cells were monocytes (Supplementary Fig. S4D), including tumor-associated macrophages (*Spp1^+^* and *C1q^+^* macrophage populations) which are clinically associated with immunosuppression and poor prognosis in patients with colorectal cancer (18, 19).

To directly assess the tumor-cell autonomous effects, we generated colonic tumor organoids from age-matched mice fed either control or supplemented diets. First, we analyzed DNA methylation at two generic repetitive elements: intracisternal A particle (IAP) and long interspersed element-1 (Line1; Fig. 2C). While methylation levels of IAP were similar between the two diets, Line1 methylation, on the other hand, was significantly reduced in tumors from supplemented mice (*P* = 0.03). This result is consistent with the known phenomenon that highly proliferative and aggressive tumors are characterized by loss of Line1 methylation (a surrogate marker of global methylation; ref. 20). We next compared the changes in promoter methylation in a panel of tumor suppressor genes that are commonly methylated in human colorectal cancer, including two Wnt regulators (*Sfrp1* and *Sfrp4*), a transcription factor (*Gata4*), a mismatch repair gene (*Mlh1*), a DNA repair enzyme (*Mgmt*), and *p16*. As shown in Fig. 2D, there was no significant difference in promoter methylation of *Sfrp1* or *Sfrp4* in mice receiving dietary supplementation compared with controls. In the *Gata4*, *Mlh1*, and *Mgmt* promoters, we found significantly increased, albeit low, levels of methylation in mice receiving dietary supplementation compared with controls. Consistent with the driver role of *p16* epimutation, we observed substantially increased *p16* promoter methylation in mice on the supplemented diet compared with controls (Fig. 2E).

## Discussion

DNA methylation as an epigenetic mechanism is functionally important because it plays a critical role in regulating gene expression. We focus on the *p16*, because, analogous to genetic mutation, *p16* epimutation is a stable change in promoter DNA methylation that occurs in a subset of aging individuals or in a subset of proliferative cells (8, 9). It is known that developmentally programmed genes, when DNA methylation is being established, are vulnerable to environmental exposures, leading to interindividual variability in methylation (21). Here, our findings expand the classic view of environmental influences on epigenetics. Importantly, we provide direct evidence that dietary nutrients can modulate age-related variations in DNA methylation, thereby serving as a natural selection mechanism for cancer-causing epigenetic regulation.

Mammals cannot make their own folate and cofactors and must obtain them from diet to produce methyl groups for DNA methylation reactions. Therefore, the ontogenic periods, when DNA methylation is being established, are vulnerable to dietary influences. Indeed, we observed increased promoter methylation following dietary supplementation at several genes that are prone to *de novo* methylation during colorectal cancer tumorigenesis. Notably, the *p16* promoter had the highest methylation and this can be explained by a combination of two reasons: increased cellular proliferation (i.e., replicative aging) and clonal expansion of cells that have *p16* methylated (i.e., selective advantage). On the other hand, our results support a model that tumor-intrinsic *p16* epimutation modulates the tumor immune microenvironment. We previously showed that reversal of epigenetic modification at the *p16* locus suppresses intestinal tumor growth and promotes durable response to immune checkpoint blockade (8). Recent studies also demonstrated that the expression of *p16* was significantly associated with senescence-associated secretory phenotype, recruitment of immune cells, and inflammatory responses (22–24). It is possible that *p16* epimutation modulates tumor immunity independent of the canonical CDK4/6-RB pathway and further work, including identification of p16 interacting proteins and single-cell and spatial transcriptomics, is still needed to clarify the role of *p16* epimutation in this process.

This work also addresses the emerging concerns regarding folic acid food fortification and colon cancer risk: one of the most common cancers and the second leading cause of cancer death in the United States. Notably, age is the biggest single risk factor for colon cancer and strongly correlated with changes in DNA methylation (25, 26). Indeed, our results show age-related *p16* promoter methylation is modulated by dietary folate and cofactors, leading to enhanced colon cancer risk. Thus, our findings highlight the need for monitoring the long-term safety of folate fortification and resulting epigenetic effects, particularly given the rising incidence of early-onset colorectal cancer in the United States over the past two decades (27).

## Supplementary Material

Figure S1 Supplementary Figure S1 shows that colon tumors from mice fed with control diet had significantly smaller size.

Figure S2 Supplementary Figure S2 shows an enhanced 1C metabolic pathway that contributes to tumor growth in response to dietary methyl donor supplementation.

Figure S3 Supplementary Figure S3 shows dietary methyl donor supplementation markedly increases tumor cell proliferation and immune cell infiltration.

Figure S4 Supplementary Figure S4 shows scRNA-seq analysis which reveals the immune landscape of colon tumors from supplemented mice.

Table S1 Supplementary Table S1 shows comparison of diets between current and previous studies.

Table S2 Supplementary Table S2 shows 78 differentially expressed metabolites in liver induced by dietary supplementation.

Table S3 Supplementary Table S3 shows 25 differentially expressed metabolites in serum induced by dietary supplementation.

Table S4 Supplementary Table S4 shows 18 differentially expressed metabolites in tumor samples induced by dietary supplementation.
