# Supplementary material for: Dietary Folate and Cofactors Accelerate Age-dependent p16 Epimutation to Promote Intestinal Tumorigenesis
Source: Cancer Res Commun. 2024 Jan 19;4(1):164–9. doi: 10.1158/2767-9764.CRC-23-0356 (PMC10798135; doi:10.1158/2767-9764.CRC-23-0356)
Supplement: Figure S1 — Supplementary Figure S1 shows that colon tumors from mice fed with control diet had significantly smaller size. [file crc-23-0356-s01.pdf]

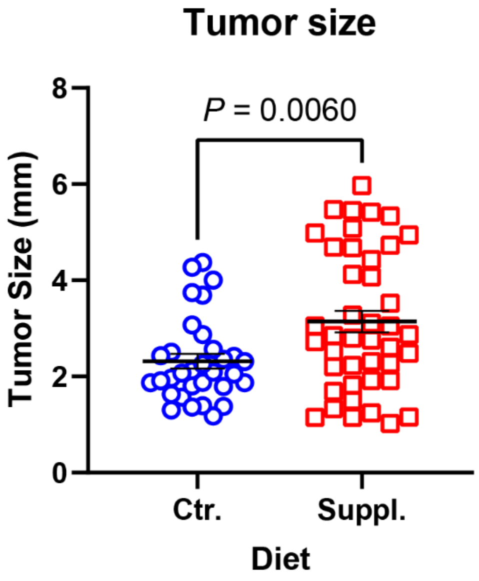

Supplementary Figure S1. Colon tumors are significantly larger in mice under dietary methyl donor supplementation. We analyzed the 15 wk-old mice fed with either control NIH-31 diet (N=5) or supplemented NIH-31 diet (N=7). We measured the diameter of individual colon tumors under a Nikon dissecting microscope using NIS-Elements software (RRID:SCR_014329). *P* value was determined by a two-tailed Student's *t*-test.
