# Supplementary material for: Dietary Folate and Cofactors Accelerate Age-dependent p16 Epimutation to Promote Intestinal Tumorigenesis
Source: Cancer Res Commun. 2024 Jan 19;4(1):164–9. doi: 10.1158/2767-9764.CRC-23-0356 (PMC10798135; doi:10.1158/2767-9764.CRC-23-0356)
Supplement: Figure S2 — Supplementary Figure S2 shows an enhanced 1C metabolic pathway that contributes to tumor growth in response to dietary methyl donor supplementation. [file crc-23-0356-s02.pdf]

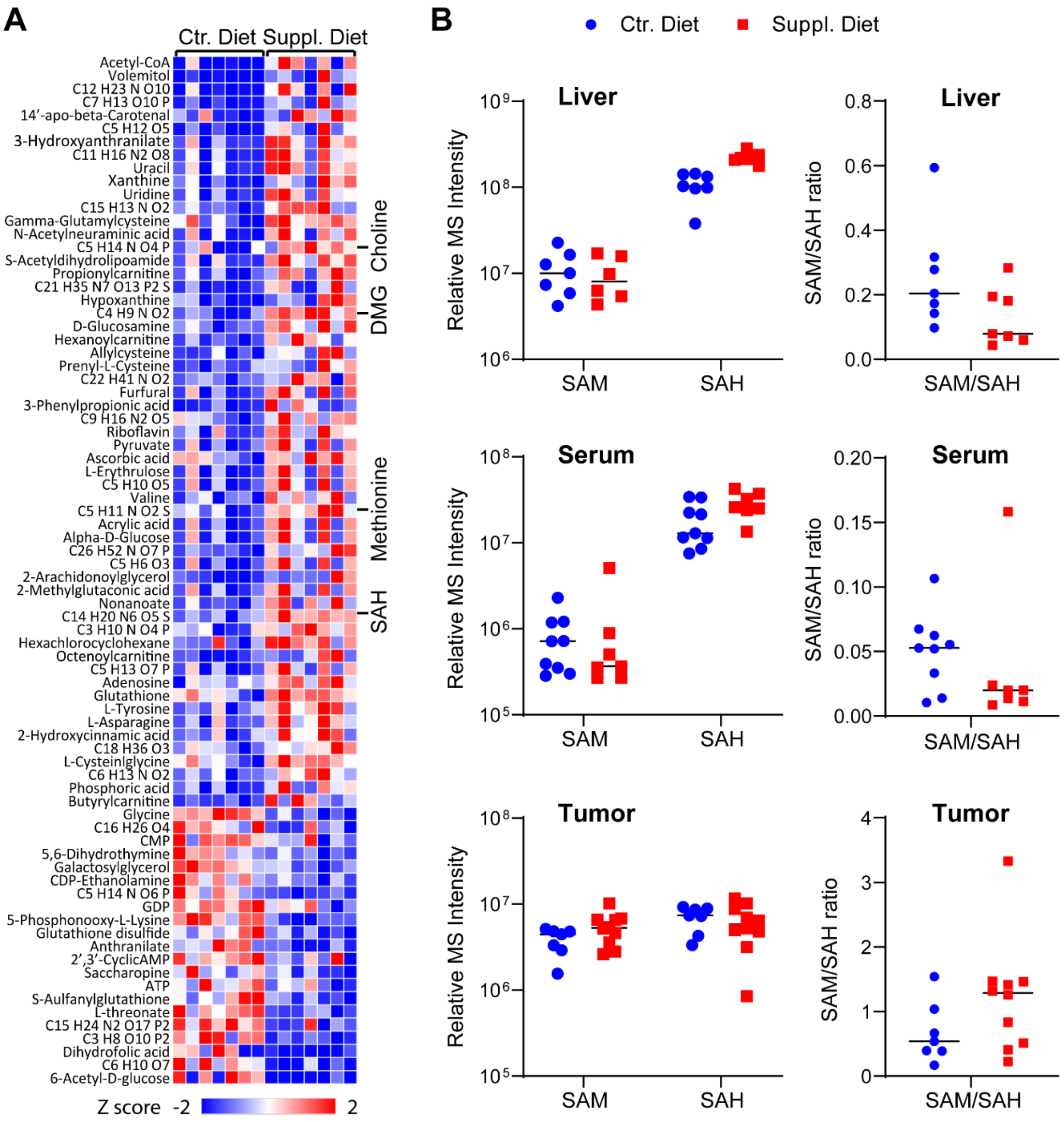

Supplementary Figure S2. An enhanced 1C metabolic pathway contributes to tumor growth in response to dietary methyl donor supplementation. **A.** Heatmap of the changes in liver metabolites related to dietary supplementation. Data were normalized using z scores to represent the relative metabolite abundances. Compared to controls, supplemented mice display multiple significantly increased 1C metabolites. **B.** Concentrations of SAM, SAH, and the SAM:SAH ratio (SAM/SAH) across all three tissue types analyzed. Increased SAM/SAH ratio was found specifically in colon tumors from supplemented mice.
