# Supplementary material for: Dietary Folate and Cofactors Accelerate Age-dependent p16 Epimutation to Promote Intestinal Tumorigenesis
Source: Cancer Res Commun. 2024 Jan 19;4(1):164–9. doi: 10.1158/2767-9764.CRC-23-0356 (PMC10798135; doi:10.1158/2767-9764.CRC-23-0356)
Supplement: Figure S3 — Supplementary Figure S3 shows dietary methyl donor supplementation markedly increases tumor cell proliferation and immune cell infiltration. [file crc-23-0356-s03.pdf]

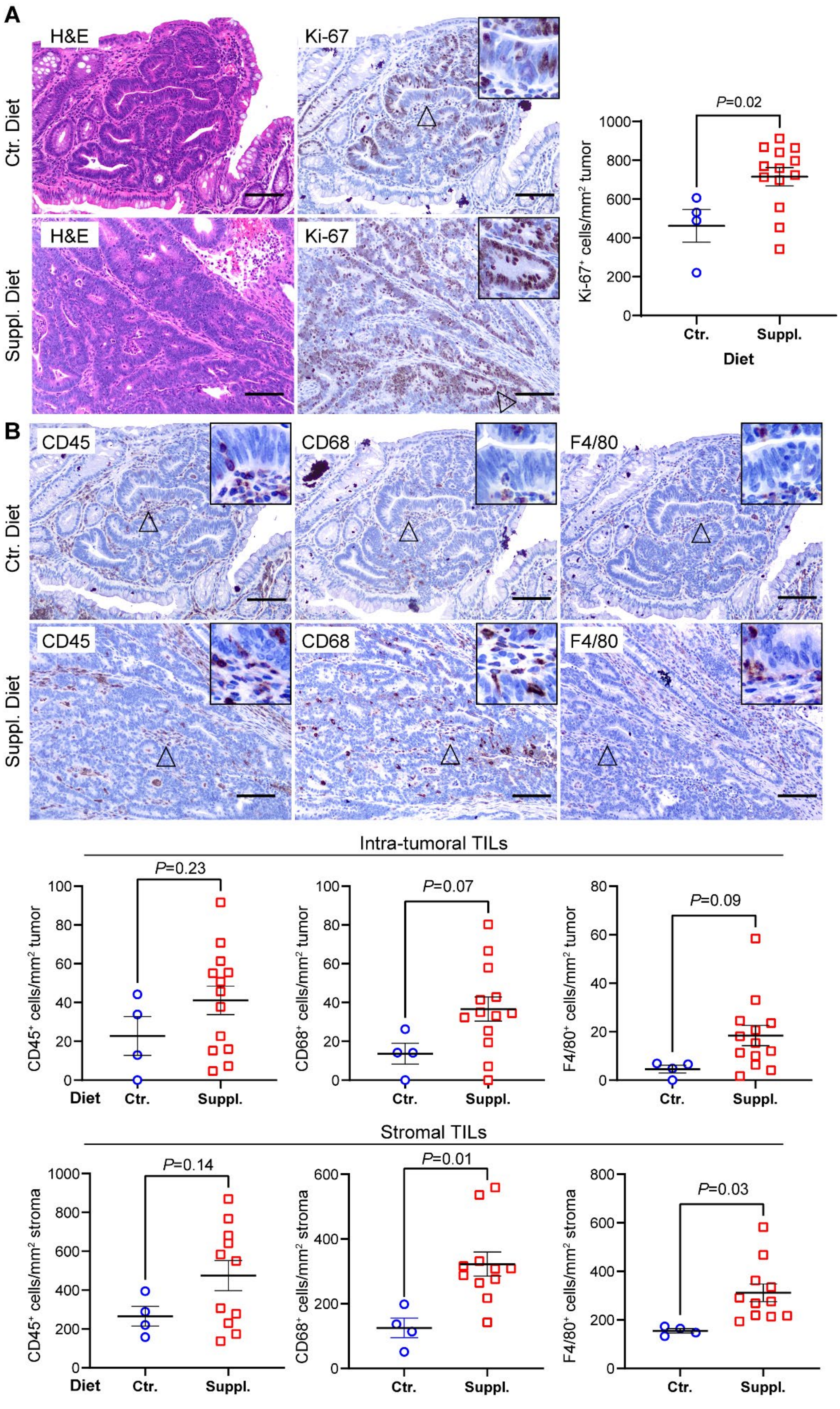
A
H&E
Ki-67
Ctr. Diet
Suppl. Diet
P=0.02
Ki-67+ cells/mm2 tumor
Ctr.
Suppl.
Diet
B
CD45
CD68
F4/80
Intra-tumoral TILs
P=0.23
P=0.07
P=0.09
CD45+ cells/mm2 tumor
CD68+ cells/mm2 tumor
F4/80+ cells/mm2 tumor
Stromal TILs
P=0.14
P=0.01
P=0.03
CD45+ cells/mm2 stroma
CD68+ cells/mm2 stroma
F4/80+ cells/mm2 stroma

Supplementary Figure S3. Dietary methyl donor supplementation markedly increases tumor cell proliferation and immune cell infiltration in the stroma. For representative images, the dietary group is indicated on the left. For immunohistochemistry (IHC) staining, a magnified view is inserted at the top right corner to show positive staining at the indicated area (open triangle). Scale bars =50 μm. For quantitative comparisons, each open dot or square represents the average number of positive cells per square millimeter (mm^2) in a tumor. **A.** IHC of Ki-67 as a proliferation marker in colon tumors. Representative images of H&E and Ki-67 staining are shown. Compared to the control group, colon tumors from mice in the supplemented group had significantly higher numbers of Ki-67$^+$ tumor cells. **B.** Profiling of tumor-infiltrating lymphocytes (TILs) by IHC. On the top, representative images show the presence of CD45$^+$ (left), CD68$^+$ (middle), and F4/80$^+$ (right) TILs within the colon tumors. Bottom panels show no statistical differences in the numbers of CD45$^+$ TILs between the two dietary groups. However, significantly increased tumor stromal infiltration of CD68$^+$ and F4/80$^+$ macrophages was observed in the mice under dietary methyl donor supplementation.
