# Supplementary material for: Dietary Folate and Cofactors Accelerate Age-dependent p16 Epimutation to Promote Intestinal Tumorigenesis
Source: Cancer Res Commun. 2024 Jan 19;4(1):164–9. doi: 10.1158/2767-9764.CRC-23-0356 (PMC10798135; doi:10.1158/2767-9764.CRC-23-0356)
Supplement: Figure S4 — Supplementary Figure S4 shows scRNA-seq analysis which reveals the immune landscape of colon tumors from supplemented mice. [file crc-23-0356-s04.pdf]

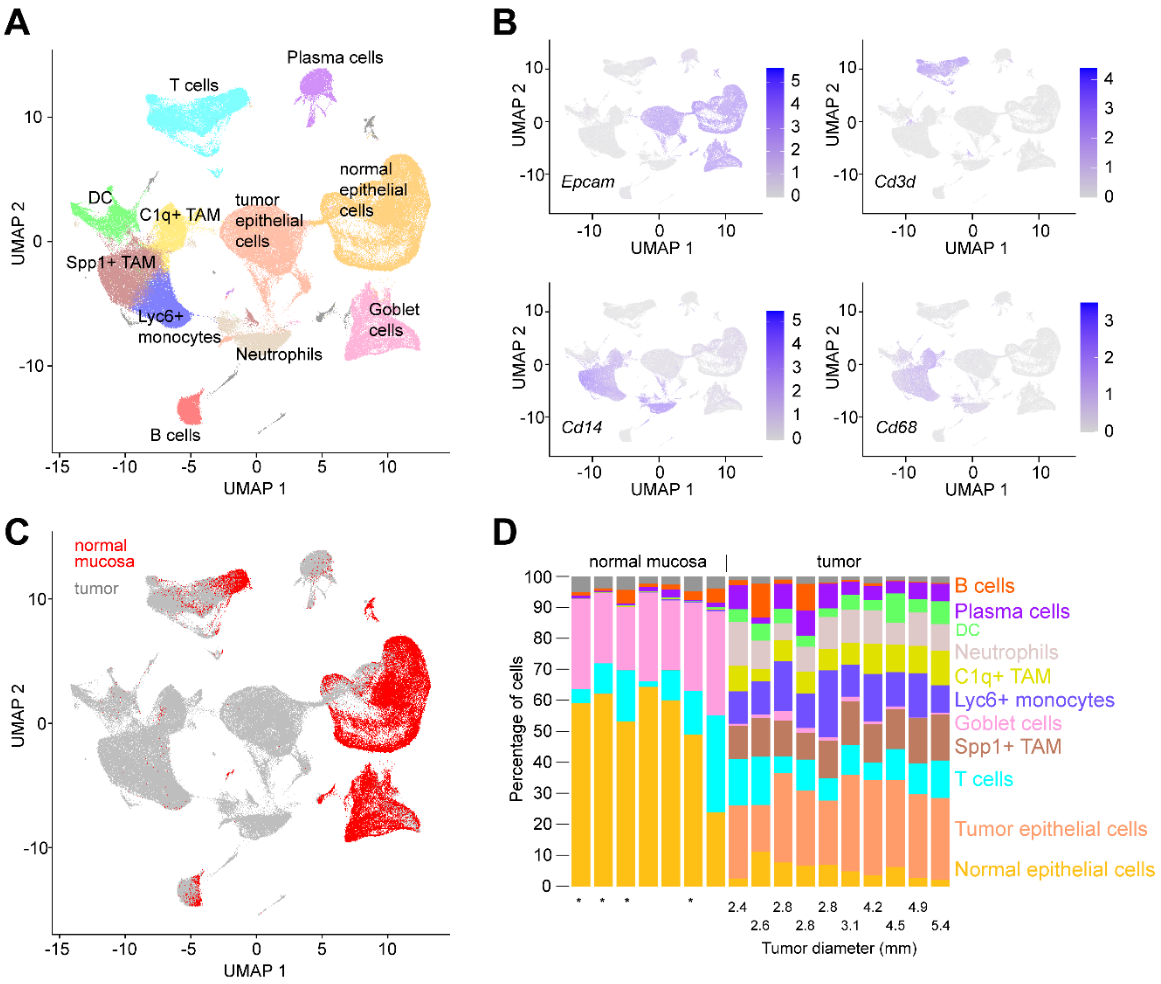

Supplementary Figure S4. scRNA-seq reveals the immune landscape of colon tumors from supplemented mice. **A.** UMAP plot showing major cell populations identified from colon tumors and adjacent normal mucosa by canonical cell markers. **B.** UMAP feature plots showing expression patterns for key marker genes, including *Epcam* (epithelial cells), *Cd3d* (T cells), *Cd14* (monocytes and neutrophils), and *Cd68* (macrophages). **C.** UMAP plot revealing distinct immune cell populations identified from tumors compared to normal tissues. **D.** A bar plot of the proportion of each cell type in individual tumors and adjacent normal colon mucosa from mice with dietary supplementation. Individual tumors are ordered by their sizes (maximum diameter). As additional controls, we included the normal mucosa samples from the previous study (Ref 8 and indicated by *).
