# Supplementary material for: Dietary Folate and Cofactors Accelerate Age-dependent p16 Epimutation to Promote Intestinal Tumorigenesis
Source: Cancer Res Commun. 2024 Jan 19;4(1):164–9. doi: 10.1158/2767-9764.CRC-23-0356 (PMC10798135; doi:10.1158/2767-9764.CRC-23-0356)
Supplement: Table S1 — Supplementary Table S1 shows comparison of diets between current and previous studies. [file crc-23-0356-s05.pdf]

Supplementary Table S1: Comparison of diets between current and previous studies

	Current NIH-31 based diet		Previous study*
	**Ctr. Diet**	**Suppl. Diet**	**LabDiet 5V5R**
Fat, %	4.47	4.47	7.6
Calcium, %	1.06	1.06	1
Vitamin D3, IU/g	4.19	4.19	2.3
Vitamin B12, mg/kg	0.06	0.5	0.08
Phosphorus, %	0.92	0.92	0.6
Fiber, %	4.05	4.05	2.4
Folic Acid, mg/kg	1.7	5	3.6
DL-methionine, %	0.39	0.39	0.6
Betaine, g/kg	2	5	NA
Choline, g/kg	1.96	5	2.2

* The standard diet (LabDiet 5V5R) was used in previously published study (Yang et al. *J Exp Clin Cancer Res* 2023; 42(1):113, PMID: 37143122). Compared to the control NIH-31 diet, the 5V5R diet is low in fiber and vit. D3 and rich in fat, folic acid, and methionine.
