# Supplementary material for: Dietary Folate and Cofactors Accelerate Age-dependent p16 Epimutation to Promote Intestinal Tumorigenesis
Source: Cancer Res Commun. 2024 Jan 19;4(1):164–9. doi: 10.1158/2767-9764.CRC-23-0356 (PMC10798135; doi:10.1158/2767-9764.CRC-23-0356)
Supplement: Table S2 — Supplementary Table S2 shows 78 differentially expressed metabolites in liver induced by dietary supplementation. [file crc-23-0356-s06.pdf]

Supplementary Table S2: 78 differentially expressed metabolites in liver induced by dietary supplementation

		Control Diet							Supplemented Diet								
**Name**	**Formula**	Ctr-1	Ctr-2	Ctr-3	Ctr-4	Ctr-5	Ctr-6	Ctr-7	MS-1	MS-2	MS-3	MS-4	MS-5	MS-6	MS-7	**FoldChange**	***P* value**
6-Acetyl-D-glucose	C8 H14 O7	28051934	33894275	43598103	2319867	31520887	1872915	41111952	248736	10257282	264194	881643	204326	911702	267647	0.01	0.01
3-dehydro-L-gulonate/D-glucuronate/L-iduronate	C6 H10 O7	24219416	28397396	37661478	19275560	51351961	18208037	51866960	2985937	12485163	1868432	23455931	2777761	1946675	4382959	0.11	0.00
Dihydrofolic acid	C19 H21 N7 O6	594886	515544	4113029	5636329	1562267	3691113	3393705	1241694	506232	416334	512907	608172	477708	879170	0.15	0.03
1,3BPG /2,3BPG	C3 H8 O10 P2	5058897	4853947	1463602	6058778	6362992	3110330	4714707	738564	282534	1303341	1895334	791989	1810570	619830	0.16	0.00
UDP-D-glucose/UDP-D-galactose	C15 H24 N2 O17 P2	30195886	22637196	38790304	24711151	36416375	17511857	35905009	8019059	13857998	1864700	35500621	6695994	5095451	6557874	0.22	0.01
L-threonate	C4 H8 O5	413247534	374273550	334943626	253619446	324019594	187764316	445763657	119782325	68258176	201948460	191391684	108693245	109755891	128586316	0.36	0.00
S-sulfanylglutathione	C10 H17 N3 O6 S2	5780205	5672259	4085348	2434563	3304984	1804164	3376156	820305	1580331	1196638	2738352	1117189	2345646	2593901	0.47	0.01
ATP	C10 H16 N5 O13 P3	66272654	52553860	39360862	35524335	75613539	28195297	43992488	14888004	9730331	22886447	54962742	34269992	42124285	22037900	0.52	0.04
Saccharopine	C11 H20 N2 O6	16957638	13078142	8149491	14340613	11103101	24153957	12906031	4910431	6831172	6505173	12793832	5931758	13896634	10624104	0.52	0.04
2',3'-Cyclic AMP	C10 H12 N5 O6 P	2847140	2382660	2110068	1730201	2025157	1353362	2608571	422833	2829332	940592	1896729	803524	1344054	1107439	0.52	0.05
anthranilate	C7 H7 N O2	273980608	546555261	510718839	620087672	347187135	372648103	333413174	199419204	334693695	182573078	186219198	173912294	283038857	271969935	0.54	0.01
GSSG	C20 H32 N6 O12 S2	410960813	356757843	257217454	196105512	262334506	158997541	234525135	87662686	138274450	96589791	201581402	106070421	226213743	171573673	0.54	0.01
5-Phosphonooxy-L-lysine	C6 H15 N2 O6 P	2368128	2316170	1584188	1999025	2496128	2634301	2178212	1276159	1300392	1345716	1019925	990085	1163028	1503606	0.55	0.00
GDP	C10 H15 N5 O11 P2	9399478	8103887	5689242	7379728	7625647	5014604	7344714	3007673	597844	2762673	7783522	4786132	5811719	4274786	0.58	0.01
sn-glycero-3-Phosphoethanolamine	C5 H14 N O6 P	96905311	111584104	148114579	174572622	110302913	147098037	205344206	78767235	105105948	86516939	84447876	69126328	87161650	91159245	0.59	0.01
CDP-ethanolamine	C11 H20 N4 O11 P2	5331764	8108820	5393138	7982106	5948834	7871257	9718777	3814660	5709871	2989396	4737194	4596991	6153874	4687999	0.60	0.01
Galactosylglycerol	C9 H18 O8	5958097	7127797	7814382	8766701	8465023	10227524	9334798	5545567	4451473	3356137	5079923	4249801	6410484	6107442	0.60	0.00
5,6-dihydrothymine	C5 H8 N2 O2	164107752	199355575	173789230	247286905	257960503	243124944	313331251	130588744	154372439	108047693	172403016	148256719	205889129	150005164	0.62	0.01
CMP	C9 H14 N3 O8 P	5066285	6135465	6353874	5791130	6305742	3295206	7113071	3066978	4156741	2122876	6832202	3801803	3815756	4352937	0.62	0.04
3-oxo-8(R)-hydroxy-hexadeca-6E,10Z-dienoate/3-oxo-8(S)-(	C16 H26 O4	2850053	1541684	1853261	2063533	2433385	2231506	2760342	1126768	1798829	1519341	2520437	1287115	1234952	1410493	0.63	0.02
Glycine	C2 H5 N O2	187430313	214315558	226594050	237425257	191263226	203330671	192732372	111630814	133922683	109342158	148181926	124650976	169946760	130140734	0.64	0.00
butyrylcarnitine	C11 H21 N O4	978071455	728457275	1165579024	908916749	902416977	1093237977	869667792	1386475230	1030557172	1242850015	1870488400	2417617674	1135863487	2327980284	1.53	0.02
Phosphoric acid	H3 O4 P	663487442	661346218	579473492	964086666	583584552	977567615	779571613	1127707656	1018028891	1306821091	737280430	776516611	1547580865	995669050	1.53	0.03
L-isoleucine/L-leucine	C6 H13 N O2	409746325	400578242	246803499	622943821	388919466	559167722	390562376	492853043	595653768	991754611	887707067	617168064	726249999	498621815	1.54	0.01
L-cysteinylglycine	C5 H10 N2 O3 S	1169743	3981458	9387018	15502301	13051841	23242954	16432501	15692702	19916621	24505996	23920486	20398477	30321053	20306744	1.56	0.01
(R)-10-Hydroxystearate/(R)-2-Hydroxystearate	C18 H36 O3	5462251	3201680	5725786	7869832	8102210	9695892	5956178	11435089	14381429	9391022	9541371	7612334	8793729	8139339	1.58	0.02
2-Hydroxycinnamic acid	C9 H8 O3	10648723	6329619	4736485	13113596	6998087	8876566	7401965	7813374	16598818	18604366	11789195	11904206	18220441	10285926	1.61	0.02
L-asparagine	C4 H8 N2 O3	46353229	46950590	41624102	75978086	34536062	60825598	44581980	61450602	75000142	98689128	68329559	58366279	99842789	75730079	1.62	0.01
L-Tyrosine	C9 H11 N O3	90974518	71654069	48439692	111317911	64173451	81249122	64479524	82000033	144252399	154646283	116070904	102430985	149772421	115369705	1.62	0.00
glutathione	C10 H17 N3 O6 S	264318307	856197297	2258890208	3001801828	2480944706	4124798341	3021448478	2988285144	3564836150	4397382168	4325044462	3644315083	5277251811	4057594336	1.64	0.01
adenosine	C10 H13 N5 O4	310565739	407977418	254292625	362201363	342996645	433182983	121090045	366724877	686519827	591934355	340105329	594905764	561047006	432875732	1.64	0.01
2-C-Methyl-D-erythritol 4-phosphate	C5 H13 O7 P	1373806281	1128216967	1074383720	1779754079	1090266719	2159313976	1325606594	2261116599	1453961464	2499725368	1294786640	1798727640	2874398852	2175834686	1.64	0.04
octenoylcarnitine	C15 H27 N O4	3856472	2455327	1846365	1883861	1640730	3400307	3104676	3439098	10126113	8914916	5346405	3016640	3040159	4053461	1.65	0.04
alpha-Hexachlorocyclohexane	C6 H6 Cl6	7179961	4615103	5962608	11331141	6034089	6198215	6585411	9886457	6571296	11175010	9304058	10270774	11969402	11599170	1.66	0.01
N-Methylethanolamine phosphate	C3 H10 N O4 P	1271050199	450494454	394055852	265017480	1169376214	863536145	1027943664	1488968942	1041198639	1433191564	2028453984	1715096879	923978193	1305894952	1.66	0.01
S-Adenosylhomocysteine	C14 H20 N6 O5 S	59078612	51659073	20483232	60372191	40041714	58273012	38190294	90353339	87002427	99511137	87490692	85326669	123865562	86909894	1.69	0.00
nonanoate	C9 H18 O2	80286105	53501367	65377333	68463167	62406673	95167193	64032773	79776488	133057028	81203283	111011950	64261710	127806527	110922250	1.70	0.02
2-methylglutaconic acid	C6 H8 O4	186822175	146416664	126196095	236185957	134536626	306795181	167447201	314532816	182152153	361685745	171149956	228349920	388601932	291425597	1.74	0.05
2-Arachidonoylglycerol	C23 H38 O4	1120128	621782	770147	674487	1253547	1596487	1917232	4592498	6402408	1725799	1907864	2026974	1543278	2312116	1.81	0.04
2-Hydroxy-2,4-pentadienoate	C5 H6 O3	348339191	267181644	263144233	425236244	274140744	597682637	312661606	580250872	340513552	702406414	322538067	459173171	753877434	592579955	1.86	0.04
1-Oleoylglycerophosphocholine	C26 H52 N O7 P	84076438	106652325	147035215	129704804	141183001	190103174	118932298	348988968	379303091	188755005	241062903	194890202	171129751	252578814	1.86	0.01
alpha-D-glucose	C6 H12 O6	630167919	487530758	455533519	856638471	494831957	1083437820	582812397	1103306763	629948521	1337170627	580004551	888213448	1404468085	1090512468	1.87	0.04
Acrylic acid	C3 H4 O2	393706575	335585487	308365353	487991688	340545752	649902524	356622212	670916309	386668179	796959948	378792571	532599218	821396565	668520899	1.87	0.04
L-methionine	C5 H11 N O2 S	31354400	22351428	19019346	48422259	29856767	48664737	42221223	47615215	79491297	80377495	58798502	50279710	61028607	55201996	1.88	0.00
Valine	C5 H11 N O2	66386704	200094788	180440514	66172237	289568888	226687074	101834679	183961200	541779108	338379157	218000317	396962216	261217092	468935302	1.88	0.01
L-Xylose/L-Xyluose/beta-D-Xylose/beta-D-Ribofuranose/alp	C5 H10 O5	33274870	27154170	24073437	43598278	25159454	56549556	29842966	57153096	32048632	70938342	30259085	46683189	71732669	56114446	1.88	0.04
L-erythrulose	C4 H8 O4	167685920	148964796	139894786	241508664	139718978	312107872	169072286	315658746	181250375	362991843	169956261	251477296	406830243	317013780	1.88	0.04
Ascorbic acid	C6 H8 O6	965603	409756991	992629027	943061606	1742069023	2052380978	1957711304	1826682785	2910252586	2033934318	3019308151	1002018049	1901482291	1902702643	1.92	0.04
pyruvate	C3 H4 O3	51953565	38158605	32516721	56682572	33585305	77321800	43020824	83425306	43895844	96796697	39667174	63495964	104226202	84713018	1.94	0.04
Riboflavin	C17 H20 N4 O6	3730211	3247943	3441165	7931279	2569313	5933226	4514105	6558397	7252013	10424215	5053403	5508464	9985136	8166519	1.94	0.01
gamma-L-glutamyl-L-alpha-aminobutyrate	C9 H16 N2 O5	3056731	1685725	2578275	3454266	4691128	4880914	6081471	7102701	6735449	3810994	6993801	4358073	9039276	4941235	1.95	0.02
3-Phenylpropionic acid	C9 H10 O2	3525503	3156289	2346478	6836597	3388337	3066587	2608215	5748520	3601932	4173464	8590259	12903075	6201982	15940571	1.96	0.04
Furfural	C5 H4 O2	41014228	34965398	33111119	51659667	30388315	69617692	36704539	80985666	40024924	91886255	39533740	65624952	91229134	73319906	2.00	0.02
(9Z)-1-(4-Morpholinyl)-9-octadecen-1-one	C22 H41 N O2	4445010	4503425	2085194	4514386	6149926	5173715	3634487	10743668	1811554	10004786	9448767	13541955	5581082	6395697	2.10	0.04
Prenyl-L-cysteine	C8 H15 N O2 S	967183	411106	458962	730500	220153	533350	394336	1471270	1096360	2055500	436941	619469	928179	977532	2.13	0.04
allylcysteine	C6 H11 N O2 S	1044639	2165432	1001449	3930910	1703327	1539463	1691135	2764215	7037119	6677390	1950305	3769047	4123544	3577463	2.23	0.02
Hexanoylcarnitine	C13 H25 N O4	233576580	110485950	224005183	315014848	165092346	458767750	213276776	566502577	255984279	546537567	695848053	997344362	432848540	669762421	2.53	0.01
D-Glucosamine	C6 H13 N O5	26854680	32534492	21886959	3963800	34000648	60299644	37645936	108609432	48459298	119823952	41717936	63962986	101487211	83525525	2.57	0.00
Dimethylglycine	C4 H9 N O2	122194723	51970532	43318669	50618294	106628709	124775907	83867363	212641435	151190715	270889301	278159458	207342887	253832827	217684359	2.60	0.00
Hypoxanthine	C5 H4 N4 O	275908728	328076488	295826653	593691336	279827059	883106085	851970837	1460942193	1850054888	1633352151	336920826	715873818	880170130	566852542	2.68	0.05
3'-Dephospho-coenzyme A	C21 H35 N7 O13 P2 S	397048	241407	183263	279126	175441	666026	308039	605324	1598112	758494	235016	278340	1123672	1006761	2.72	0.04
Propionylcarnitine	C10 H19 N O4	404167004	162543140	202662047	661620972	258116871	763914786	382589631	1082730517	1405960705	959323261	326589314	1056310149	1185472847	577160656	2.76	0.01
S-Acetyldihydrolipoamide	C10 H19 N O2 S2	111428638	128593370	118932655	299649650	81051292	309502292	138027265	410745013	290288619	422006223	215235289	266431555	466809120	362893868	2.82	0.00
choline phosphate	C5 H14 N O4 P	2500430437	600719679	636951834	557184127	3275313202	2016162645	1146790324	2672180003	3584433733	2767602012	4454344182	3249588108	3356131767	1455504891	2.83	0.01
N-acetylneuraminic acid	C11 H19 N O9	2914146	3388923	3229965	8495498	3218256	10165355	4259739	11895807	7182949	10950924	3516491	8275036	13667143	9879718	2.92	0.03
Gamma-Glutamylcysteine	C8 H14 N2 O5 S	1170216	1667624	4947115	11907758	4567931	18867671	11381182	15203497	18978854	15500072	14456161	12215218	22160288	19529639	3.13	0.01
N-Hydroxy-2-acetamidofluorene	C15 H13 N O2	4124038	2251236	5076786	6926923	5402253	10870534	7315277	7137251	7633600	20567487	17463790	17960122	17351552	11891520	3.21	0.01
uridine	C9 H12 N2 O6	184669668	274340123	179978549	622864218	125454458	774789726	319107392	782203227	695789715	1242175661	339321121	892827624	1408023047	1247169645	3.25	0.01
Xanthine	C5 H4 N4 O2	210719914	291400052	245023768	597585347	194169253	950963592	549450133	1404912674	1131487647	1680688852	343865658	664209762	1014847162	652141640	3.48	0.02
uracil	C4 H4 N2 O2	60036967	68417835	54739134	196118497	35179751	224010207	94290804	252700676	219564132	327914848	108711009	267867711	363546187	346680539	3.92	0.00
N-Acetylaspartylglutamic acid	C11 H16 N2 O8	6309166	6722794	5364709	22108186	3894261	24847502	9078136	25645400	21305473	39434491	10212296	26701763	44192496	42264762	3.97	0.01
3-hydroxyanthranilate	C7 H7 N O3	1339232	1550549	1318827	3571523	558208	5751775	1820029	6319418	5141046	8450756	2121069	4861189	7779297	7995214	4.08	0.00
L-arabinitol/xylitol/D-ribitol	C5 H12 O5	3920685	3121377	2623125	8534579	2937392	12919401	3620467	19261870	9739093	37235784	3980128	12503321	23049067	18169473	5.02	0.02
14'-apo-beta-Carotenal	C22 H30 O	164058	87607	230173	201733	1374226	327471	724746	1424969	1847821	704472	1331973	1858320	332213	679394	5.79	0.03
2-Dehydro-3-deoxy-D-arabino-heptonate 7-phosphate	C7 H13 O10 P	1346761	1320708	683827	4571314	354172	7809067	1586745	12116723	7642077	29278671	2342307	6065910	12919333	9817431	7.29	0.03
6-(alpha-D-Glucosaminyl)-1D-myo-inositol/Lactosamine	C12 H23 N O10	316825	331434	169227	568870	216378	3794037	237860	6807776	199054	4971939	353936	3998497	6549250	3478129	12.62	0.03
Volemitol	C7 H16 O7	130788	173950	158027	392505	109933	1066679	188455	3398821	2274050	8288648	164236	1155757	3214026	1984936	13.07	0.04
acetyl-CoA	C23 H38 N7 O17 P3 S	100158	93432	165244	87875	96549	1392291	138459	1769481	99822	1566300	349897	1726566	2317794	1118425	15.64	0.02
