# Supplementary material for: Dietary Folate and Cofactors Accelerate Age-dependent p16 Epimutation to Promote Intestinal Tumorigenesis
Source: Cancer Res Commun. 2024 Jan 19;4(1):164–9. doi: 10.1158/2767-9764.CRC-23-0356 (PMC10798135; doi:10.1158/2767-9764.CRC-23-0356)
Supplement: Table S3 — Supplementary Table S3 shows 25 differentially expressed metabolites in serum induced by dietary supplementation. [file crc-23-0356-s07.pdf]

Supplementary Table S3: 25 differentially expressed metabolites in serum induced by dietary supplementation

		Control Diet									Supplemeted Diet								
**Name**	**Formula**	Ctr1	Ctr2	Ctr3	Ctr4	Ctr5	Ctr6	Ctr7	Ctr8	Ctr9	MS1	MS2	MS3	MS4	MS5	MS6	MS7	**FoldChange**	***P* value**
L-arginine	C6 H14 N4 O2	457802458.5	165793837.2	548428481.1	166522423	189746149.5	39471415.33	318241176.2	18417538.43	109840417	84362364.23	16531486.73	92149028.2	29564792.12	36729617.19	39472146.23	141187655	**0.24**	**0.03**
anthranilate	C7 H7 N O2	313856751.3	374110657.1	397737215	343062499.1	331745584.9	595318138.5	393236549.3	463217840.1	328953120.4	192019699.1	229893852.2	142053769.6	211022349.7	194893216.6	260009431.6	268674940.1	**0.56**	**0.00**
orotate	C5 H3 N2 O4	12985224.14	5149425.06	7562416.496	7807732.448	7891627.055	11371594.96	4317858.831	4193865.283	7822707.321	28924192.76	14436098.76	29952921.16	12578312.77	9050186.689	14901743.56	10413470.86	**1.85**	**0.03**
thymine/imidazol-4-ylacetate	C5 H6 N2 O2	20466891.8	14954879.22	17012916.95	13763994.79	15663230.48	16993735.71	24823141.2	14865114.09	9890485.349	31096837.92	9457826.78	39923606.56	33045049.45	18249149.49	22035788.97	29377368.26	**1.88**	**0.05**
trans-Hexadec-2-enoyl carnitine	C23 H43 N O4	11725796.3	16593200.46	6956119.436	6205262.632	6055130.52	13967285.99	13960632.59	6260828.938	8581530.396	12172853.31	16146591.32	25936336.91	9506607.908	12424485.93	20128432.88	19441019.9	**1.88**	**0.03**
Linoleyl carnitine/Linoelaidyl carnitine	C25 H45 N O4	12092225.13	13081110.12	8487881.524	7015127.756	6671168.532	6783104.552	14292869.38	5300807.111	6422932.593	18944162.88	19894701.44	24418902.02	7707053.835	5961710.759	15086920.33	14788783.29	**2.15**	**0.05**
lactate	C3 H6 O3	28135663509	31210147266	21795621810	31956475797	36753906954	42451188399	26072737994	37283366821	30078928317	40921741786	51668369461	40915351647	47294129847	22826305401	56493444324	51518345607	**1.52**	**0.02**
succinate(2-)	C4 H4 O4 -2	2795542628	2180856599	1346953500	1757338147	2491003730	1797991109	1655667304	1581784975	1655548815	2855027728	2472646495	2732311741	3103679243	2352982730	2783523441	4546161831	**1.58**	**0.01**
Propionic acid	C3 H6 O2	211532391.9	176891110.1	107195071.5	138506793.1	191202816.7	155951099.1	136702044.9	123564025.7	121759519.1	231023100.2	194757851.1	215226328.6	261528218.3	189876682.6	237310232.6	364562427.9	**1.67**	**0.01**
D-Glucuronate 1-phosphate/D-Glucuronate 6-phosphate	C6 H11 O10 P	40863246.1	26674625.73	12109256.44	19643507.84	46270465.15	18709219.81	6143974.381	13891186.27	18896677.73	47066107.13	41714815.48	47390060.47	72121965.46	36195462.43	50656714.36	108764171.6	**2.51**	**0.01**
N6-Me-Adenosine	C11 H15 N5 O4	17335257.02	9504085.265	9870823.955	9768182.458	6643613.252	15032911.23	21161265.18	16672858.66	6443351.484	15653711.76	19938493.34	22031279.29	19790726.6	18642516.37	17091511.34	20130886.31	**2.00**	**0.01**
3-hydroxyhexadecanoylcarnitine	C23 H45 N O5	2306450.833	1751858.269	521514.8084	985473.4161	2144546.505	2497881.934	3852300.845	3706007.947	1025656.813	1368632.268	6697155.631	5307904.457	4141010.277	2024247.055	6115489.236	3016074.886	**1.93**	**0.05**
Hypoxanthine	C5 H4 N4 O	2309214968	775434915.6	518212386.8	894102917.6	1928360083	2645950442	4719744914	4036995561	648689038.4	4158954521	5708260280	5154075419	5231973765	3738496649	5846325969	2943947917	**2.67**	**0.00**
inosine	C10 H12 N4 O5	149299456.9	59331665.15	38972857.93	32275466.94	93362841.46	162865430.7	237384689.7	385951359.3	25024907.69	303441492.3	298180825.3	282003954.8	301220384.6	232977633.3	318880701	54736271.11	**3.19**	**0.03**
uracil	C4 H4 N2 O2	172835327.8	69406456.07	55904509.06	56724002.51	86589775.46	141476727.9	95234474.04	120677864.5	24412431.14	231260638.5	358947270.2	344556661.1	344316270.3	75606989.6	244413496.4	126421397.9	**2.82**	**0.01**
propionyl-carnitine	C10 H19 N O4	399869006.1	476258363.6	472370476	345593849.2	643779920.3	665309818.8	746499922	504444817.6	380468161.9	420775134.8	630121357.2	751456046.7	602892083.4	877859225.3	921153095.9	1063667030	**1.58**	**0.03**
glycine betaine	C5 H11 N O2	9315672019	9163858247	7554597787	7309762227	7954258369	12838915022	11531929586	11564105044	9612268469	14797405501	13430252183	11087868489	17089281943	16700776654	13026303450	17530803448	**1.59**	**0.00**
N,N-Dimethylglycine	C4 H9 N O2	154972577.2	121161422.3	145399481.2	103730158	107716873.6	291968803.4	169595245.1	161882300.9	156015601.7	447229676.1	253366576.4	216888452.7	569259087.6	439076990.1	344503566.7	317153280.1	**2.22**	**0.00**
Trimethylamine N-oxide	C3 H9 N O	708742185.2	575530050.7	1009020809	196607750.5	208531148.9	264807076.1	209711565.1	208332443.9	214696170.6	1343310155	608222018.9	323021167.3	1803854744	1129207320	1093737492	1224342874	**5.26**	**0.01**
Sedoheptulose 7-phosphate	C7 H15 O10 P	3791946.178	3542164.653	2761856.96	5638104.172	6115497.946	16391867.67	21164652.41	12461925.34	4551937.483	15605726.56	15450334.4	11817501.75	24105542.28	23930039.09	18942230.18	8157290.233	**2.77**	**0.02**
Phosphodimethylethanolamine	C4 H12 N O4 P	4202669.695	8805292.759	3461904.134	7763174.721	10110016.23	18623783.64	42333949.78	15144787.38	13779242.85	26945701.32	31234573.96	15213218.57	51849979.11	35744664.12	28891443.81	18045099.54	**2.86**	**0.02**
uridine	C9 H12 N2 O6	491886424	212513127.2	292889952.3	264226135.8	796923665.1	649460870.8	830347014.6	975851938.3	172570213.6	678314873.2	747373867	766194731	1538657213	1377159959	1759818156	1703860908	**2.80**	**0.01**
L-arabinitol/xylitol/D-ribitol	C5 H12 O5	12231953.39	8393174.861	7257468.23	7867617.973	18786269.38	22345676.78	35967546.13	16798160.79	9839796.656	22695517.57	46314521.54	20969148.17	49087033.96	34318946.14	44767407.91	43143823.26	**3.53**	**0.00**
L-Iditol/D-glucitol/galactitol	C6 H14 O6	14476743.4	12410366.13	11170711.89	12033638.75	22798913.98	25233415.43	31273895.48	22529082.6	10718829.46	22047174.98	53575804.41	24653180.41	58326363.69	43831513.35	52308932.31	55463912.47	**3.61**	**0.00**
xanthosine	C10 H12 N4 O6	40195238.23	10519084.87	11582902.06	21803732.77	98355328.69	76266236.49	230869309.9	171440567.3	10718655.88	94912923.81	299477255.9	115898768.4	257183843.6	213843302.5	264308023.6	190915049.9	**5.32**	**0.01**
