# Supplementary material for: Dietary Folate and Cofactors Accelerate Age-dependent p16 Epimutation to Promote Intestinal Tumorigenesis
Source: Cancer Res Commun. 2024 Jan 19;4(1):164–9. doi: 10.1158/2767-9764.CRC-23-0356 (PMC10798135; doi:10.1158/2767-9764.CRC-23-0356)
Supplement: Table S4 — Supplementary Table S4 shows 18 differentially expressed metabolites in tumor samples induced by dietary supplementation. [file crc-23-0356-s08.pdf]

Supplementary Table S4: 18 differentially expressed metabolites in tumor samples induced by dietary supplementation

	Control Diet							Supplemented Diet											
**Name**	Ctr-1	Ctr-2	Ctr-3	Ctr-4	Ctr-5	Ctr-6	Ctr-7	MS-1	MS-2	MS-3	MS-4	MS-5	MS-6	MS-7	MS-8	MS-9	MS-10	**FoldChange**	***P* value**
Triisopropanolamine	237529696.9	3087164183.0	4794531858.6	3567239444.2	4381851411.9	2793173017.7	3766647632.9	2578848034.6	173182309.4	2827303889.9	361834964.0	2922037.5	10372326.0	6762470.1	14835364.0	231246067.4	4588216149.7	**0.334**	**0.01**
Deoxyadenosine monophosphate	704851.9	539441.9	501131.1	376642.8	708445.4	638345.0	667162.0	159384.5	178016.6	197433.7	223063.4	233509.6	549017.7	393997.9	376087.0	543599.9	482128.8	**0.565**	**0.00**
UMP	14615342.4	17521296.6	21246265.7	18116554.2	13240166.7	18492248.8	24860309.1	9977534.5	7701126.8	20243922.7	6357477.8	5785352.6	10332499.7	9799780.2	4577253.6	7497467.3	12026972.0	**0.515**	**0.00**
sn-Glycero-3-phosphocholine	1320100123.6	1595017758.7	1472603166.9	1662390582.9	1755491962.9	1835456432.5	1490400121.4	902453994.1	842644976.3	450988891.0	1036072133.5	846847943.5	1290769807.3	936314110.1	440799235.4	1753095844.2	882445834.3	**0.590**	**0.00**
propionyl-carnitine	104247761.2	161742955.1	231197230.2	156409270.8	91273083.9	155862302.2	118289741.0	51903017.1	130981804.5	94726097.4	94401231.7	71138274.9	136067700.3	88227564.5	124244478.5	83337376.3	89301829.3	**0.662**	**0.03**
UDP-D-glucose/UDP-D-galactose	4919771.1	5937220.5	6687693.0	6517462.8	5644222.3	6214119.9	5756080.0	5558847.2	3262347.8	4401371.7	3067231.8	3496376.0	3960305.2	1565571.9	2034724.6	4270182.3	5645921.6	**0.626**	**0.00**
UDP	9154620.0	17609291.2	12297659.3	11603443.2	9893508.3	15082994.7	10710392.9	8120944.1	5418070.0	18420236.9	5478180.1	7959466.0	7445097.4	3325571.7	5280295.8	7755841.3	10980195.7	**0.650**	**0.03**
decanoyl carnitine	5226448.7	5132136.1	5964088.8	5040493.2	3455043.4	7863259.6	6265172.1	3519045.7	3052015.7	6135582.6	1657763.5	3566890.2	6726117.1	507717.4	4946216.0	2991259.3	3434135.5	**0.657**	**0.03**
Linolenelaidic acid	4161306.5	5355540.1	5122158.0	4970851.2	6563513.0	5957412.2	5814212.6	9759595.5	4196341.8	8251177.4	6561395.8	10210818.2	6757433.8	8732393.4	11993407.4	7884746.9	7613622.1	**1.512**	**0.00**
D-Glucosamine 6-phosphate/D-Glucosamine 1-phosphate	20742838.4	20203660.7	27959476.9	23285292.1	38416910.4	18486063.0	13543141.2	34499311.8	37884089.3	35564589.6	42287938.6	49479024.7	22743141.2	34198497.7	29172212.7	50734011.0	33727595.8	**1.594**	**0.00**
Eicosenoic acid	23084880.5	10453654.0	14985271.1	14603307.8	20858050.6	14698732.2	16229401.1	26908307.8	21309306.0	22019829.5	24544883.8	33342051.1	16053448.6	14726914.1	30607825.5	29681668.8	27273732.5	**1.501**	**0.01**
oleic acid/elaidate/trans-vaccenate	234616034.2	192854252.4	185946807.6	181739372.0	236403819.3	176991304.3	192425740.7	334038522.4	270371521.8	264360231.6	327042479.2	383704502.6	225816283.7	305063721.2	444161919.4	396052642.7	387126835.2	**1.668**	**0.00**
D-Glucuronate 1-phosphate/D-Glucuronate 6-phosphate	27299.4	27549.1	27677.7	28156.7	43160.2	26860.6	28335.2	63246.3	92255.3	27511.3	72139.4	113514.2	26139.3	26599.4	26372.2	34696.2	100583.0	**1.952**	**0.03**
N,N-Dimethylglycine	11283783.9	8316547.6	7537064.5	6766465.9	8532044.9	6712998.8	7886127.4	13637001.1	15211665.2	13031247.3	15323799.7	18078773.6	12589996.1	14690161.0	13002672.8	16471279.9	15424344.6	**1.810**	**0.00**
Riboflavin	312704.8	122794.7	474854.8	560038.4	511336.6	246599.0	140169.2	911993.7	965462.8	324313.7	839356.7	1419976.4	194411.7	236944.7	485336.1	1037202.6	664066.0	**2.092**	**0.02**
Tetracosatetraenoyl carnitine	116405.9	77131.5	126335.5	97022.9	139665.8	69048.0	76285.2	374556.1	940351.7	141078.8	383399.3	619009.4	103657.1	134297.1	294301.6	272098.0	212392.3	**3.466**	**0.01**
